# Crocins from *Crocus sativus* L. in the Management of Hyperglycemia. In Vivo Evidence from Zebrafish

**DOI:** 10.3390/molecules25225223

**Published:** 2020-11-10

**Authors:** Eleni Kakouri, Adamantia Agalou, Charalabos Kanakis, Dimitris Beis, Petros A. Tarantilis

**Affiliations:** 1 Laboratory of Chemistry, Department of Food Science & Human Nutrition, School of Food and Nutritional Sciences, Agricultural University of Athens, 111855 Athens, Greece; elenikakouri@aua.gr (E.K.); chkanakis@aua.gr (C.K.); 2 Developmental Biology, Biomedical Research Foundation Academy of Athens, 11527 Athens, Greece; agalou@bioacademy.gr

**Keywords:** crocins, glucose, β-pancreatic cells, insulin, *pck1*

## Abstract

Diabetes mellitus is a disease characterized by persistent high blood glucose levels and accompanied by impaired metabolic pathways. In this study, we used zebrafish to investigate the effect of crocins isolated from *Crocus sativus* L., on the control of glucose levels and pancreatic β-cells. Embryos were exposed to an aqueous solution of crocins and whole embryo glucose levels were measured at 48 h post-treatment. We showed that the application of crocins reduces zebrafish embryo glucose levels and enhances insulin expression. We also examined whether crocins are implicated in the metabolic pathway of gluconeogenesis. We showed that following a single application of crocins and glucose level reduction, the expression of *phosphoenolpyruvate carboxykinase* *1* (*pck1*), a key gene involved in glucose metabolism, is increased. We propose a putative role for the crocins in glucose metabolism and insulin management.

## 1. Introduction

Diabetes mellitus (DM) is a chronic metabolic disease characterized by persistent high blood glucose levels and accompanied by impaired metabolic pathways of carbohydrates, proteins, and lipids. Diabetes is essentially caused either by the loss of β-cells, or of their ability to produce insulin (type I). Type II diabetes refers to the inability of the organism to properly regulate and sense insulin, known as resistance to insulin. Hyperglycemia is the result of both types. Since insulin is the main transporter, responsible for removing glucose from the blood-stream, its deficiency can inescapably lead to elevated blood glucose levels. Persistent hyperglycemia can lead to severe micro and macrovascular complications causing long-term damage to nerves and blood vessels, affecting different body organs. In this regard, the management of blood glucose levels in diabetic patients is the main focus of the available antidiabetic treatment including either administration of insulin and/or synthetic drugs [1].

Numerous medicines have been developed over the past years to alleviate symptoms, increase life expectancy, and maintain the progression of the disease in remission. Medical treatments through synthetic formulations can overcome risk factors. There are several cases where synthetic formulations lead to severe side effects that are not associated with the disease itself. In order to avoid treatment related side effects, scientists are driven towards the development of new therapeutic molecules, able to replace current therapeutic strategies.

Phytochemicals are emerging as powerful alternatives towards the fight against chronic diseases, including diabetes. These plant-derived molecules used as a mixture or as a single compound, contribute to the prevention and treatment of several chronic disorders such as cancer, neurodegenerative and metabolic diseases. It has been proposed that their efficacy is mainly due to their antioxidant activity [2,3]. Treatment with natural products is yet preliminary and much information is still needed regarding their mechanism of action. These include pharmacological parameters such as pharmacokinetics and pharmacodynamics, drug tolerance, and possible side effects. Accumulating data of several in vitro, in vivo, and clinical trial studies provide evidence that these substances are of great therapeutic importance and may consist a new era of treatment against the above-mentioned long-term conditions [4,5,6].

*Crocus sativus* L. is a stemless, perennial plant belonging to the Iridaceae family and the genus Crocus. The plant is commonly known as saffron, a name which is referred to its dried red stigmas. Stigmas are considered the pharmacologically active part of the plant and their chemical composition has been widely studied [7,8,9,10,11]. More than 150 volatile and non-volatile compounds have been identified. Crocins (CRCs) are the predominant constituents of the stigmas and give them their characteristic deep red color [10,11,12,13]. On the contrary to other carotenoids, CRCs due to their glycosylated terminals, are water soluble molecules.

Zebrafish, *Danio rerio*, a small tropical fish, has become a popular model for studying a wide range of human diseases. These include cancer [14], cardiovascular [15], neurodegenerative [16] and metabolic diseases [17,18]. This is due to the high genetic homology with humans and the similarities in organ physiology and metabolism, offering several unique advantages. Among those, most useful for this study are the external fertilization, development, and transparency of the embryos allowing non-invasive in vivo imaging, as well as the plethora of synchronized progeny. In vivo phenotypic screens using zebrafish embryos have been particularly valuable in identifying novel bioactive natural compounds or optimizing the activity of lead compounds [19]. Chemical library screens using zebrafish embryos for the identification of melanogenesis inhibitors [20] and or transgenic-based screens for angiogenesis [21] or neoglucogenesis [18] have been very productive.

In addition, zebrafish has become a popular experimental model regarding the study of metabolic diseases, including diabetes. Similarities regarding the exocrine and endocrine pancreas between mammals and zebrafish [22], the conservation of key proteins that control glucose metabolism [18], the capability of measuring glucose levels in larvae and adult zebrafish, as well as the unique capacity of this model to recover its β-cells [23,24], make zebrafish a promising experimental model for testing compounds that alter glucose metabolism or regulate glucose levels [25,26].

We aimed to investigate the effect of CRCs isolated from *Crocus sativus* L., on the control of glucose levels using zebrafish as an animal model. We also examined whether the addition of crocins would regulate the levels of *phosphoenolpyruvate carboxykinase 1* (*pck1*), a key regulatory gene for the gluconeogenesis process that contributes to the maintenance of normoglycemia. Finally, we evaluated the effect of crocins on β-pancreatic cells.

## 2. Results

### 2.1. LC-QTOF HRMS Analysis

Although the chemical profile of CRCs is well documented in previous studies [10,11,12,13], we evaluated the quality of the fresh prepared extract used in this study. Crocins are found in the extract of *Crocus sativus* L. stigmas conjugated with different types of sugars. Identification of the types of CRCs presented was performed by the LC/Q-TOF/HRMS analysis. The quadrupole time-of-flight tandem mass spectrometer (Q-TOF/MS) is a hybrid analyzer, as it couples a TOF instrument with a quadrupole instrument. Therefore, on the contrary to conventional HPLC-MS systems, LC/Q-TOF/MS offers more accurate results since Q-TOF/MS, not only provide the chemical formula of a compound based on accurate mass measurement (mass error less than 5 ppm), but also high resolution and high detection sensitivity make this technique, a powerful tool for precise analysis of a mixture. The compounds presented in Table 1 confirm previous studies [10,11,12,13], as five types of CRCs were tentatively identified.

### 2.2. Determination of LC50

We used zebrafish larvae to evaluate the effect of crocins on glucose metabolism in vivo during embryo development. In order to avoid toxic effects for the treated embryos from the CRCs application, a toxicity test was performed before proceeding with the biological experiments. LC50 was estimated according to the OECD guidelines [27] as described in the Materials and Methods Section and it was calculated at 0.681 mg/mL in an extract of crocins used in this study (Table 1).

### 2.3. Zebrafish Glucose Levels Are Lowered by CRCs

**Figure 1:**
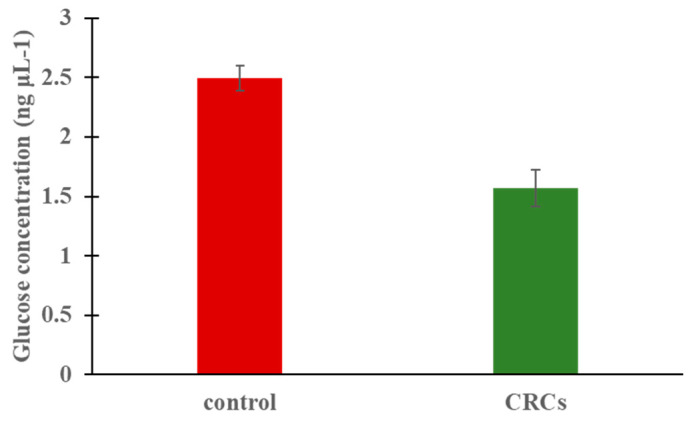
Glucose levels at zebrafish larvae following 48h treatment with CRCs at the concentration of 0.2 mg/mL. Data are mean +/− standard error of the mean (SEM), n = 3, *p* < 0.05.

To investigate whether the treatment of crocins can regulate glucose levels of zebrafish embryos, larvae at 72 h post fertilization (hpf) were treated with 0.2 mg/mL CRCs for 48 h. This concentration corresponds to approximately 1/3 of the calculated LC50 and is considered safe since no effects in terms of mortality or any abnormalities were detected in the developing embryos. Three independent experiments were performed and the results showed that there was a significant decrease (*p* < 0.05) of glucose levels on treated embryos compared with the untreated embryos (Figure 1), indicating that the application of crocins can affect glucose levels.

### 2.4. Enhanced Fluorescence of β-Cells Indicate Insa Upregulation

**Figure 2:**
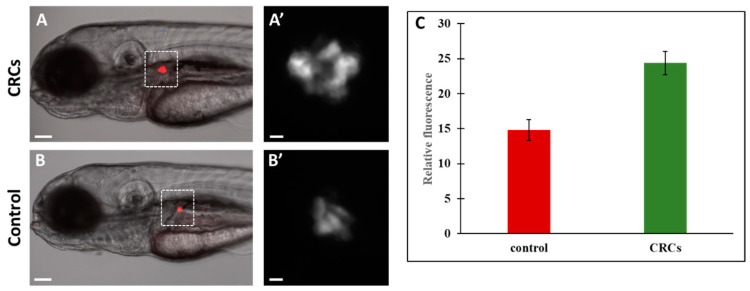
Zebrafish pancreas monitoring after treatment with CRCs. Transgenic zebrafish *Tg*(*ins:DsRed*) were treated at 72hpf with 0.2 mg/mL CRCs for 48h and visualized under fluorescent microscope. There was significant increase of fluorescence on the pancreatic islets of CRCs-treated embryos (**A**,**A′**) compared to the control group (**B**,**B′**). **A’** and **B’** are micrographs of **A** and **B**, respectively. (**C**) fluorescence intensity was quantified using Fiji software. Results shown are the mean +/− SEM. *p* < 0.01. Scale bars: A and B: 100 μm, A’ and B’:10 μm.

Since pancreatic β-cells secrete insulin to regulate glucose metabolism, we aimed to investigate the effect of CRCs on the β-cell formation of developing zebrafish embryos. For this purpose, we used the transgenic zebrafish line *Tg*(*ins:DsRed*), where the expression of the red fluorescent protein is driven by the zebrafish prepro-insulin promoter providing a convenient fluorescent marker for β-cells. Zebrafish embryos at 72 hpf were treated with CRCs and after 48 h of incubation the insulin-expressing cells of the pancreatic islets were visualized under a fluorescent microscope. A significant increase in fluorescent intensity was observed on the CRCs-treated embryos compared to the control group (Figure 2). Since the fluorescence of β-cells in this transgenic line is driven by the insulin reporter, these results indicate that the application of crocins promoted insulin expression.

### 2.5. Insulin Expression by Quantitative Real-Time PCR

**Figure 3:**
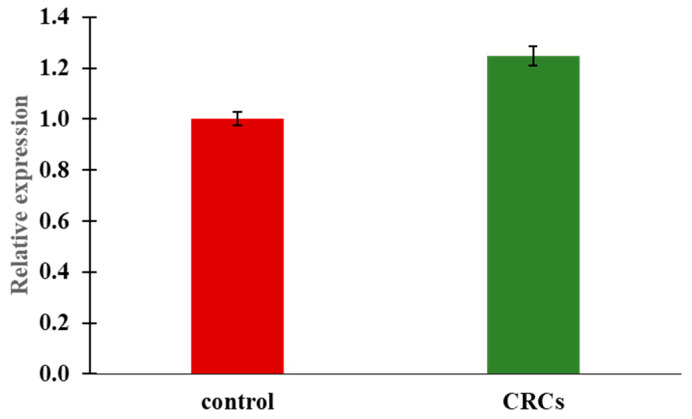
Expression of *insa* following 48h treatment with CRCs at the concentration of 0.2 mg/mL. *Insa* is significantly upregulated compared to the control treated embryos whose gene expression was set as 1. mRNA expression was normalized against *ef1a*. Data are mean +/− standard error of the mean (SEM), n = 3, *p* < 0.01.

In order to confirm that the enhanced fluorescence of the zebrafish pancreatic islets is the result of increased endogenous insulin expression, rather than that of the transgene only, we determined the levels of insulin mRNA by RT-PCR. Three insulin genes have been discovered in zebrafish, namely insulin a (*insa*), insulin b (*insb*), and insulin c (*insc*). *Insa* and *insb* are expressed as early as 1 hpf indicating that both genes are maternally expressed [28,29]. However, *insa* reaches its peak at 72 hpf, a time point in which an almost mature zebrafish pancreas is established, on the contrary to *insb,* whose expression is restricted only during very early developmental stages. In addition, *insa* is expressed specifically at the pancreas, whereas *insb* is expressed also in the brain. Therefore, it has been hypothesized that *insa* is the prominent responsible gene for glucose homeostasis regulation [30]. *Insc* is quite a newly discovered gene, expressed in the gut and internal organs [29], but its exact function is still not clear. In this respect, we focused on the study of *insa.* In addition, the promoter elements of this gene were also used for the generation of the transgene. The expression of *insa* gene were evaluated using qRT-PCR on 120 hpf zebrafish larvae treated at 72 hpf for 48 h with 0.2 mg/mL of CRCs. Our data showed that after the administration of CRCs expression of *insa,* it was significantly upregulated compared to the control group (*p* < 0.01 (Figure 3), supporting the implication of crocins in glucose homeostasis.

### 2.6. Pck1 Expression Induced as a Response to Lower Glucose Levels

**Figure 4:**
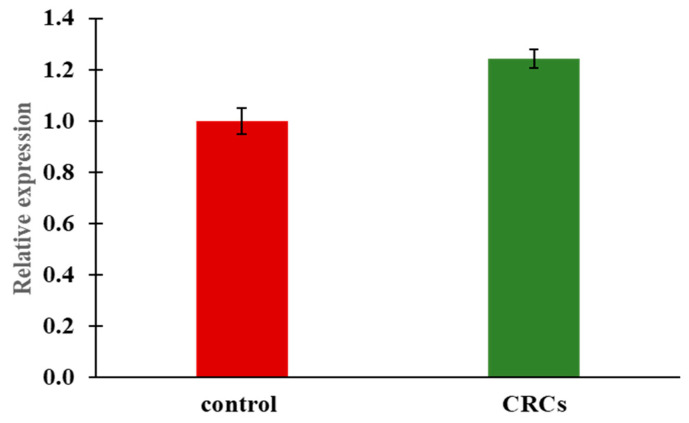
Expression of *pck1* following 48h treatment with CRCs at the concentration of 0.2 mg/mL. *Pck1* is significantly upregulated compared to the control treated embryos whose gene expression was set as 1. mRNA expression was normalized against *ef1a*. Data are mean +/− standard error of the mean (SEM), n = 3, *p* < 0.0001.

*Pck* is one of the main genes involved in glucose metabolism and is transcriptionally regulated among others by insulin. It can be found in the cytosol (*pck1*) and the mitochondria (*pck2*). However, only the cytosolic isoform, expressed in the liver, is responsible for catalyzing the formation of phosphoenolpyruvate from oxaloacetate and is predominantly implicated in the gluconeogenesis pathway [30]. In order to uncover the mechanism of implication of CRCs on glucose regulation we evaluated the expression of *pck1* gene using RT-PCR at 48 hpt. Our results showed that treatment of the zebrafish embryos with crocins lead to an induction of *pck1* following the reduced glucose levels (Figure 4). These data indicate that the reduction of glucose on CRCs-treated zebrafish larvae is not via the inhibition of the gluconeogenesis pathway (*pck1*), but rather induce a homeostatic response.

## 3. Discussion

*Crocus sativus* L., is a plant with rich pharmacologic activities. It has been studied in the context of several diseases such as cancer, neurodegenerative disorders, inflammation, heart related, and metabolic disorders. These properties are mainly attributed to the stigmas of the plant and specifically to its main constituents, crocins (CRCs), picrocrocin, and safranal [31,32,33,34].

However, growing, harvesting, storage, and environmental conditions, in addition to the extraction procedure, are important criteria to consider for the quality of the raw material. In herbal medicine, the quality of the material is directly linked to the efficacy of the natural product when tested in either in vivo or in vitro experiments and has a high impact on the study results. However, environmental conditions are rather uncontrolled and thus, a quality control test is always needed in order to guarantee the presence of the desired compounds. Furthermore, storage conditions including temperature, humidity, and light strongly affect the stability of the compounds presented. The above-mentioned quality criteria for the raw material used in this study are followed identically for each harvesting crop.

Saffron safety has been evaluated in clinical trials and in vivo studies [35,36,37]. Generally, saffron extracts as well as its constituents are relatively safe. For example, the intraperitoneal administration of stigmas ethanolic extract in rats at a range of a concentration between 1–5 g/kg bw (body weight), caused lethal effects at the highest administered dose (100% mortality), whereas the lowest dose resulted in no death at the end of 48 h. Authors estimated the LD50 value at 3.5 g kg^−1^ bw [35]. Similarly, Hosseinzadeh et al., (2010) [36] examined the safety of an extract of CRCs in mice and rats, after oral or intraperitoneal administration. No toxic effect was stated by the authors at mice treated with the extract since no mortality was observed at 2 h and after 48 h of treatment. The oral administrated dose was 3 g/kg, while the intraperitoneal injection was within the range of 0.5–3 g/kg. According to these data, stigmas of *Crocus sativus* L., and its active constituent CRCs, show a safe profile, since, an LD50 value within the range of 1–5 g/kg is attributed to low-toxic chemicals according to the toxicity classification [38].

In our experiments, the administration of CRCs is performed via immersion of the embryos in an aqueous solution of the tested compound. Even though the exact amount of crocin uptake is not known, this is a standard method for toxicity evaluation of compounds in zebrafish. Since, to our knowledge, this is the first time that a purified extract of crocins is tested in zebrafish, we investigated a range of concentrations up to 2 mg/mL in order to evaluate the toxicity. To investigate the effect of crocins on the control of glucose levels, we performed all the experiments at a concentration that corresponds to 1/3 of the LC50, and did not result in any phenotypic abnormalities. In this way, we are ensured that all measurements are not affected by any toxic response of the developing larvae.

The antidiabetic and hypoglycemic potential of *Crocus sativus* L., is supported by many studies and saffron is nowadays considered as a promising candidate in the field of metabolic diseases. For example, Kianbakht and Hajiaghaee, 2011 [39] discussed the anti-glycemic effect of crocins, safranal, and saffron extracts in diabetic rats. Their results indicated that these compounds managed to control glycemia without triggering any toxic effect in the liver or the kidney. Another study performed by Kang et al., 2012 [40] demonstrated the in vitro capacity of saffron extract to activate glucose uptake and ameliorate insulin sensitivity in skeletal muscle cells. Furthermore, Dehghan et al., 2016 [41] showed the effectiveness of saffron to improve diabetic biochemical markers, such as blood glucose and glycosylated hemoglobin, in in vivo and in vitro models. In addition, several clinical trials have confirmed the antidiabetic effect of saffron in type II diabetes patients. Milajerdi et al., 2018 [42] performed a triple-blinded randomized clinical trial in type II diabetic patients and suggested that saffron extracts, administrated twice a day and used in combination with current antidiabetic therapy, lowered blood glucose levels. In addition, the administration of saffron in overweight/obese patients diminished both sugar levels and hemoglobin A1c [43,44].

Streptozotocin-induced diabetic models have been used to evaluate the effect of CRCs on glucose levels and pancreatic function. Rajaei et al., 2013 [45] evaluated serum glucose levels of hyperglycemic rats treated intraperitoneally with crocins. Lower glucose levels observed in the study with respect to the control group were attributed to higher insulin production or to the strong antioxidant activity of the extract used. The latter antidiabetic mechanism was further investigated by Yaribeygi et al., 2019 [46], who measured SOD and catalase levels of pancreas in diabetic rats. In this study, they found that CRCs significantly increased both enzymes and thus, harmful effects caused by oxidative stress due to hyperglycemia were avoided.

In another study, the hypoglycemic and hypolipidemic effects of CRCs were evaluated in streptozotocin-induced type II diabetes rats, where advanced glycation products, glucose and HbA1c levels, and fasting insulin levels were measured. All these parameters were significantly decreased in treated animals [47].

Despite the fact that zebrafish is widely used as a model for many human disorders, including metabolic diseases, there are only few studies analyzing the effect of saffron and/or its bioactive compounds on glucose management. In our study, we used zebrafish embryos to investigate the effect of the main bioactive compounds of *Crocus sativus* L. on the regulation of the glucose levels and the insulin secretion. We found that the administration of CRCs can significantly reduce glucose levels and in parallel increase insulin expression in developing embryos, suggesting a putative hypoglycemic role of this compound. Our study used a single application of CRCs in the water of developing embryos and more studies including adult feeding with CRCs would be needed to address long-term effects in zebrafish glucose homeostasis.

In general, various mechanisms have been proposed based on histopathologic observations and on measurement of blood glucose levels in several in vitro experimental models to explain the hypoglycemic and antidiabetic activity of saffron. Among them, enhanced stimulation of insulin secretion from β-cells, increased capacity of the peripheral tissues to use it properly, restoration of the β-cells of the endocrine pancreas, and inhibition of glucose production. In addition, the antioxidant activity of the stigmas seems to drastically influence its hypoglycemic effect [45,48,49]. Here, we present elevated fluorescence levels of the pancreatic islets in the *Tg*(*ins:DsRed*) transgenic fish and increased transcription of the endogenous insulin gene as a result of CRCs treatment of zebrafish larvae. These observations support the hypothesis that the hypoglycemic activity of crocins is at least in part, due to the enhanced insulin production of β-cells.

*Pck1* is a key gene in the process of gluconeogenesis. It is often used as an indicator of blood glucose levels since it is normally downregulated in cases of hypoglycemia and upregulated when blood glucose levels are elevated. In our study, lowering the levels of glucose were linked with a slight but significant increase in *pck1* expression. This could be explained as a homeostatic, compensatory response of the zebrafish larvae to the reduced glucose levels induced by CRCs. Nevertheless, glucose production is simultaneously related to stimulated metabolism, an issue that needs to be further investigated taking into account that diabetes patients suffer metabolism complications. The same effect of low glucose levels and overexpression of *pck1* was also in the *Tg(pck1:Luc2*, *cryaa:mCherry)^s952^* bioluminescence transgenic zebrafish line [18]. Authors tested several chemical compounds for their capacity to reduce glucose levels and also studied their effect on *pck1* expression. Among the tested compounds PK1195, a translocator protein 18 kDA (TSPO) ligand, induced *pck1* expression simultaneously to a reduction in glucose levels. Authors concluded that compounds that belong to the TPSO family, regardless of their gluconeogenetic effect, can effectively interfere with levels of glucose in zebrafish larvae.

In summary, we employed zebrafish as a tool in order to explore the effect of crocins isolated from *Crocus sativus* L. on glucose metabolism. Our results suggest that the treatment of zebrafish larvae with non-toxic levels of crocins can significantly reduce the basal total glucose levels and increase insulin expression. These data provide some evidence that crocins are implicated in the glucose regulation, but further studies are needed in order to elucidate the exact mechanism of action and its potential as a putative agent on the antidiabetic field.

## 4. Materials and Methods

### 4.1. Extraction and Chemical Analysis of Crocins

Stigmas of *Crocus sativus* L. were cultivated at the Kozani prefecture and were kindly offered by the “Cooperative of Saffron producers, Kozani Greece”. Stigmas were collected in 2018.

*Crocus sativus* L. is a male-sterile triploid lineage that ever since its origin has been propagated vegetatively. No wild population has been found so far and all the available plant material comes from cultivation. The crop is an autotriploid that evolved in Attica by combining two different genotypes of *C. cartwrightianus* (a species endemic to Attica and some Aegean islands, Greece) [50]. Triploid sterility and vegetative propagation prevented afterwards segregation of the favorable traits of saffron, resulting in worldwide cultivation of a unique clonal lineage. As a result, no infraspecific taxa have been recognized.

Five grams of dried stigmas were shattered and extracted with petroleum ether in order to remove lipid compounds, in an ultrasonic water bath (GRANT type, 300 × 140 × 150 mm internal dimensions), for 15 min at 25 °C and at the frequency of 35 kHz. The extraction was repeated 5 times and proceeded under the same conditions using diethyl ether. Extraction with petroleum ether and diethyl ether proceed prior to methanol extractions in order to eliminate the final extract from the presence of unwanted compounds such as lipids and safranal. The received powder was dried under nitrogen steam and the final extraction step took place as described above, using methanol. The organic solvent was evaporated in a rotary evaporator and a purified extract of CRCs was received. The final product that consisted of a red colored powder was kept at −4 °C for further analysis.

In order to ensure total evaporation of the organic solvent, an aqueous extract of CRCs was prepared and Raman spectroscopy was performed. No characteristic peaks that correspond to the presence of methanol were observed, indicating that the final product was methanol free.

The analysis of the extract was performed on an HPLC system (Agilent Series 1260-Agilent Technologies, Waldbronn, Germany) coupled to a 6530 Q-TOF mass spectrometer (Agilent Technologies, Singapore). The HPLC system consists of degasser, autosampler, quaternary pump, diode array detector, and a thermostatically controlled column oven. Chromatographic separation was performed at 40 °C on a Poroshell 120 EC-C18 4.6 × 50 mm, 2.7 μm reversed phase column. The following chromatographic conditions were applied: Flow rate 0.4 mL/min, injection volume 5 μL, mobile phase A (water LC/MS-0.1% formic acid), and mobile phase B (acetonitrile LC/MS-0.1% formic acid). The gradient elution program was applied as follows: 5–95% solvent B from 0 to 33 min and maintained at 95% up to 38 min. The sample was detected at 440 nm. The Q-TOF mass spectrometer was operated with a dual ESI source in the negative ionization mode and according to the following operating parameters: Capillary voltage 4000 V, gas temperature 300 °C, skimmer 65 V, octapole RF 750 V, drying gas 10 L/min, nebulizer pressure 450 psig, and fragmentor voltage 150 V. Scanning was performed from 100–1700. The Q-TOF-MS was calibrated on a daily basis using a reference mass solution (calibrant solution, Agilent Technologies) with internal reference masses at *m*/*z* 112.9856 and 1033.9881. The data acquisition and qualitative analysis were processed by MassHunter software (Agilent Technologies).

### 4.2. Determination of LC50

The toxicity of crocins on zebrafish embryos was evaluated according to OECD guidelines (OECD 2013 Toxicity test TG236, 2013). Briefly, fertilized embryos were collected at 3 hpf and treated with an aqueous solution of CRCs extract. A range of concentrations from 0–2 mg/mL was tested. The concentration of 0 mg/mL corresponds to the control group.

During the experiment the test media was not renewed, embryos were kept at 28 °C, and the plate was covered with aluminum foil to protect the crocins from degradation. Embryos were monitored daily up to 96 hpf and scored for lethality Three replicates were performed for each concentration tested. Results were calculated using the probit analysis (IBM SPSS Statistics v23). In order to ensure the stability of the extract, extracts of the same concentration were stored under the same conditions, and their UV-Vis spectra were recorded daily. The extracts remained stable during the experiment (data not shown).

### 4.3. Zebrafish Maintenance and Breeding

Zebrafish were raised under standard laboratory conditions at 28 °C on a 14/10 h day/night cycle in the animal facility of Biomedical Research Foundation of the Academy of Athens (EL25BIO003). Zebrafish are maintained according to the Recommended Guidelines for Zebrafish Husbandry Conditions [51]. The genetic backgrounds used were the wild-type Ab strain for all toxicity, glucose level quantification and RT-PCR experiments, and the transgenic line *Tg*(*ins:DsRed*) for visualizing pancreatic β-cells. The experimental protocols described in this study were completed by day 5 of the zebrafish embryo development and therefore, are not subject to the regulations of European animal protection guidelines, in accordance with the European Directive 2010/63 for the protection of animals used for scientific purposes.

### 4.4. Measurement of Glucose Levels

The measurement of glucose levels was performed on larvae zebrafish using a colorimetric/fluorimetric based enzymatic detection kit (Biovision-K606) and according to Jurczyk et al., 2011 [22]. Zebrafish larvae at 72 hpf were placed in a 12-well plate and treated with the extract of crocins at a concentration of 0.2 mg/mL. Embryos were incubated at 28 °C for 48 h. The treatment was performed in triplicate.

The measurement of glucose was performed according to the protocol provided with slight modifications. Briefly, 20 embryos were placed in eppendorf tubes and the medium was removed. The collected embryos were frozen in liquid nitrogen and were kept at −80 °C for a minimum of 30 min. Then, 80 μL of phosphate-buffered saline (PBS) were added in each tube and embryos were homogenized using a pestle. Tubes were centrifuged and collection of the supernatant took place 8 μL of each sample were added in 42 μL of PBS in a 96-well plate. After that, 50 μL of the reaction mix containing one μL of glucose probe, one μL of glucose enzyme, and 48 μL of glucose buffer were added so as to adjust the final volume at 100 μL. To calculate the results, a standard curve of glucose was constructed (0–3.5 nmol/well). Reactions were incubated at 37 °C for 30 min and absorbance was measured at 570 nm.

### 4.5. RNA Isolation and cDNA Synthesis

Total RNA extraction from 120 hpf zebrafish larvae was carried out using TRIzol reagent (15596026-Invitrogen) and purification was performed with Turbo DNase (2238G2-Ambion), according to the manufacturers’ protocols. The RNA concentration and purity were determined by NanoDrop 2000c Spectrophotometer (Thermo Scientific, Waltham, MA, USA) and cDNA was synthesized using the PrimeScript RT reagent kit (RR037A-Takara).

### 4.6. Quantitative Real-Time PCR

RT-PCRs were performed with a Roche cycler system (Light Cycler 96) using the KAPA SYBR FAST qPCR kit (KK4611-KAPA Biosystems) and gene-specific primers. The sequences of primers used are the following:

*pck1f*: TCTCCATCCCTCCGCTCATCA, *pck1r*: GGCCCAGCTGACTGCTCCT, *insaf*: TAAGCACTAACCCAGGCACA, *insar*: GATTTAGGAGGAAGGAAACC.

As a reference gene, the elongation factor was used with the following primers: *efa1f:* TCTCTACCTACCCTCCTCTTGGTC and *efa1r*: TTGGTCTTGGCAGCCTTCTGTG. The relative amounts of the different mRNAs were quantified with the ΔΔCt method [52] and the fold-change ratio was calculated and expressed as mean ± SEM.

### 4.7. Monitoring Pancreas Development

Zebrafish larvae from the transgenic line *Tg*(*ins:DsRed*) were treated with 0.2 mg/mL of CRCs at 72 hpf. Embryos were incubated at 28 °C for 48 h and subsequently anesthetized using 0.4 mg/mL of Tricaine (BIA 1347-Apollo scientific) in order to proceed to imaging. To facilitate imaging, the embryos were mounted in a 1.2% low melting agarose. Fluorescent and brightfield images were captured using a Leica DMRA2 microscope (Leica, Switzerland) equipped with a HamamatsuORCA-Flash 4.0 V2 camera and analyzed using the Fiji software. Data are presented as mean ± SEM. Differences were analyzed using the two-tailed Student’s *t*-test. In addition, *p* was considered significant when less than 0.05.

## Figures and Tables

**Table 1:** Identified compounds at the negative ionization mode.

t_R_	Compound Name *	Chemical Formula	*m*/*z* Theoretical	*m*/*z* Observed [M − H]^−^	% of CRCs	Δm
10.658	Crocin 5 (*trans* 5GGG)	C_50_H_74_O_29_	1137.4230	1137.4218	0.73	−1.05
13.677	Crocin 5 (cis 5GGG)	C_50_H_74_O_29_	1137.4230	1137.4220	1.34	−0.88
14.971	Crocin 4 (*trans* 4GG)	C_44_H_64_O_24_	975.37148	975.3707	46.84	−0.80
15.047	Crocin 2 (*trans* 2G)	C_32_H_44_O_14_	651.26583	651.2639	29.30	−2.96
15.402	Crocin 4 (*cis* 4GG)	C_44_H_64_O_24_	975.37148	975.3699	14.96	−1.62
15.783	Crocin 3 (*trans* 3Gg)	C_38_H_54_O_19_	813.31865	813.317	18.29	−2.02
16.062	Crocin 3 (*cis* 3Gg)	C_38_H_54_O_19_	813.31865	813.3172	78.09	−1.78
16.645	Crocin 1 (*trans* 1g)	C_26_H_34_O_9_	489.21301	489.2117	4.84	−2.68
18.142	Crocin 2 (*cis* 2G)	C_32_H_44_O_14_	651.26583	651.2639	5.61	−2.96

* Nomenclature of CRCs followed that proposed by Carmona et al. (2006) [8]. (G) is referred to gentiobiose and (g) to glucose.

## References

[1] Choudhury H., Pandey M., Hua C.K., Mun C.S., Jing J.K., Kong L., Ern L.Y., Ashraf N.A., Kit S.W., Yee T.S. (2017). An update on natural compounds in the remedy of diabetes mellitus: A systematic review. J. Tradit. Complement. Med..

[2] Sarikurkcu C., Kakouri E., Sarikurkcu T.R., Tarantilis P.A. (2019). Study on the chemical composition, enzyme inhibition and antioxidant activity of Ziziphora taurica subsp. cleonioides. Appl. Sci..

[3] Prince P.S.M., Kannan N.K. (2006). Protective effect of rutin on lipids, lipoproteins, lipid metabolizing enzymes and glycoproteins in streptozotocin-induced diabetic rats. J. Pharm. Pharmacol..

[4] Al-Ishaq R.K., Abotaleb M., Kubatka P., Kajo K., Büsselberg D. (2019). Flavonoids and Their Anti-Diabetic Effects: Cellular Mechanisms and Effects to Improve Blood Sugar Levels. Biomolecules.

[5] Eid H.M., Haddad P.S. (2017). The Antidiabetic Potential of Quercetin: Underlying Mechanisms. Curr. Med. Chem..

[6] Ghorbani A. (2017). Mechanisms of antidiabetic effects of flavonoid rutin. Biomed. Pharmacother..

[7] Caballero-Ortega H., Pereda-Miranda R., Riveron-Negrete L., Hernandez J.M., Medécigo-Ríos M., Castillo-Villanueva A., Abdullaev F.I. (2004). Chemical composition of saffron (*Crocus sativus* L.) from four countries. Acta Hortic..

[8] Carmona M., Zalacain A., Sanchez A.M., Novella J.L., Alonso G.L. (2006). Crocetin esters, picrocrocin and its related compounds present in *Crocus sativus* stigmas and Gardenia jasminoides fruits. Tentative identification of seven new compounds by LC-ESI-MS. J. Agric. Food Chem..

[9] Ríos J.L., Recio M.C., Giner R.M., Máñez S. (1996). An Update Review of Saffron and its Active Constituents. Phytother. Res..

[10] Tarantilis P.A., Tsoupras G., Polissiou M. (1995). Determination of saffron (*Crocus sativus* L.) components in crude plant extract using high-performance liquid chromatography-UV-visible photodiode-array detection-mass spectrometry. J. Chromatogr. A.

[11] Mohajeri S.A., Hosseinzadeh H., Keyhanfar F., Aghamohammadian J. (2010). Extraction of crocin from saffron (*Crocus sativus*) using molecularly imprinted polymer solid-phase extraction. J. Sep. Sci..

[12] Karkoula E., Angelis Koulakiotis N.S., Gikas E., Halabalaki M., Tsarbopoulos A., Skaltsounis A.L. (2018). Rapid isolation and characterization of crocins, picrocrocin, and crocetin from saffron using centrifugal partition chromatography and LC-MS. J. Sep. Sci..

[13] Kanakis C.D., Daferera D.J., Tarantilis P.A., Polissiou M.G. (2004). Qualitative determination of volatile compounds and quantitative evaluation of safranal and 4-hydroxy-2,6,6-trimethyl-1-cyclohexene-1-carboxaldehyde (HTCC) in Greek saffron. J. Agric. Food Chem..

[14] Letrado P., de Miguel I., Lamberto I., Díez-Martínez R., Oyarzabal J. (2018). Zebrafish: Speeding Up the Cancer Drug Discovery Process. Cancer Res..

[15] Giardoglou P., Beis D. (2019). On Zebrafish Disease Models and Matters of the Heart. Biomedicines.

[16] Bandmann O., Burton A.E. (2010). Genetic zebrafish models of neurodegenerative diseases. Neurobiol. Dis..

[17] Zang L., Maddison L.A., Chen W. (2018). Zebrafish as a Model for Obesity and Diabetes. Front. Cell Dev. Biol..

[18] Gut P., Baeza-Raja B., Andersson O., Hasenkamp L., Hsiao J., Hesselson D., Akassoglou K., Verdin E., Hirschey M.D., Stainier Y.R.D. (2012). Whole-organism screening for gluconeogenesis identifies activators of fasting metabolism. Nat. Chem. Biol..

[19] Wiley D.S., Redfield S.E., Zon L.I. (2017). Chemical screening in zebrafish for novel biological and therapeutic discovery. Methods Cell Biol..

[20] Papakyriakou A., Kefalos P., Sarantis P., Tsiamantas C., Xanthopoulos K.P., Vourloumis D., Beis D. (2014). A zebrafish in vivo phenotypic assay to identify 3-aminothiophene-2-carboxylic acid-based angiogenesis inhibitors. Assay Drug Dev. Technol..

[21] Agalou A., Thrapsianiotis M., Angelis A., Papakyriakou A., Skaltsounis A.L., Aligiannis N., Beis D. (2018). Identification of Novel Melanin Synthesis Inhibitors from Crataegus pycnoloba Using an in Vivo Zebrafish Phenotypic Assay. Front. Pharmacol..

[22] Jurczyk A., Roy N., Bajwa R., Gut P., Lipson K., Yang C., Covassin L., Racki W.J., Rossini A.A., Phillips N. (2011). Dynamic glucoregulation and mammalian-like responses to metabolic and developmental disruption in zebrafish. Gen. Comp. Endocrinol..

[23] Curado S., Anderson R.M., Jungblut B., Mumm J., Schroeter E., Stainier D.Y. (2007). Conditional targeted cell ablation in zebrafish: A new tool for regeneration studies. Dev. Dyn..

[24] Anderson R.M., Bosch J.A., Goll M.G., Hesselson D., Dong P.D., Shin D., Chi N.C., Shin C.H., Schlegel A., Halpern M. (2009). Loss of Dnmt1 catalytic activity reveals multiple roles for DNA methylation during pancreas development and regeneration. Dev. Biol..

[25] Elo B., Villano C.M., Govorko D., White L.A. (2007). Larval zebrafish as a model for glucose metabolism: Expression of phosphoenolpyruvate carboxykinase as a marker for exposure to anti-diabetic compounds. J. Mol. Endocrinol..

[26] Seth A., Stemple D.L., Barroso I. (2013). The emerging use of zebrafish to model metabolic disease. Dis. Model Mech..

[27] OECD (2013) Test No. 236: Fish Embryo Acute Toxicity (FET) Test, OECD Guidelines for the Testing of Chemicals, Section 2. Home Page. https://www.oecd-ilibrary.org/environment/test-no-236-fish-embryo-acute-toxicity-fet-test_9789264203709-en.

[28] Papasani M.R., Robison B.D., Hardy R.W., Hill R.A. (2006). Early developmental expression of two insulins in zebrafish (Danio rerio). Physiol. Genom..

[29] Irwin D.M. (2019). Duplication and diversification of insulin genes in ray-finned fish. Zool. Res..

[30] Koren D., Palladino A., Roy E., Samuel Refetoff W. (2016). Hypoglycemia, in Genetic Diagnosis of Endocrine Disorders.

[31] Hatziagapiou K., Kakouri E., Lambrou G.I., Bethanis K., Tarantilis P.A. (2019). Antioxidant Properties of *Crocus sativus* L. and Its Constituents and Relevance to Neurodegenerative Diseases; Focus on Alzheimer’s and Parkinson’s Disease. Curr. Neuropharmacol..

[32] Azimi P., Ghiasvand R., Feizi A., Hosseinzadeh J., Bahreynian M., Hariri M., Khosravi-Boroujeni H. (2016). Effect of cinnamon, cardamom, saffron and ginger consumption on blood pressure and a marker of endothelial function in patients with type 2 diabetes mellitus: A randomized controlled clinical trial. Blood Press.

[33] Pitsikas N., Tarantilis P.A. (2018). Effects of the active constituents of *Crocus sativus* L. crocins and their combination with memantine on recognition memory in rats. Behav. Pharmacol..

[34] Ayatollahi H., Javan A.O., Khajedaluee M., Shahroodian M., Hosseinzadeh H. (2014). Effect of *Crocus sativus* L. (saffron) on coagulation and anticoagulation systems in healthy volunteers. Phytother. Res..

[35] Mohajeri D., Mousavi G., Mesgari M., Doustar Y., Nouri M.H.K. (2007). Subacute Toxicity of *Crocus sativus* L. (Saffron) Stigma Ethanolic Extract in Rats. Am. J. Pharmacol. Toxicol..

[36] Hosseinzadeh H., Sadeghi Shakib S., Khadem Sameni A., Taghiabadi E. (2013). Acute and subacute toxicity of safranal, a constituent of saffron, in mice and rats. Iran. J. Pharm. Res. IJPR.

[37] Mehri S., Razavi B.M., Hosseinzadeh H. (2020). Safety and Toxicity of Saffron, in Saffron.

[38] (2004). United States Environmental Protection Agency, Chemical Hazard Classification and Labeling: Comparison of OPP Requirements and the GHS: Draft. https://www.epa.gov/sites/production/files/2015-09/documents/ghscriteria-summary.pdf.

[39] Kianbakht S., Hajiaghaee R. (2011). Anti-hyperglycemic Effects of Saffron and its Active Constituents, Crocin and Safranal, in Alloxan-Induced Diabetic Rats. J. Med. Plants..

[40] Kang C., Lee H., Jung E.S., Seyedian R., Jo M., Kim J., Kim J.S., Kim E. (2012). Saffron (*Crocus sativus* L.) increases glucose uptake and insulin sensitivity in muscle cells via multipathway mechanisms. Food Chem..

[41] Dehghan F., Hajiaghaalipour F., Yusof A., Muniandy S., Hosseini S.A., Heydari S., Salim L.Z., Azarbayjani M.A. (2016). Saffron with resistance exercise improves diabetic parameters through the GLUT4/AMPK pathway in-vitro and in-vivo. Sci. Rep..

[42] Milajerdi A., Jazayeri S., Hashemzadeh N., Shirzadi E., Derakhshan Z., Djazayeri A., Akhondzadeh S. (2018). The effect of saffron (*Crocus sativus* L.) hydroalcoholic extract on metabolic control in type 2 diabetes mellitus: A triple-blinded randomized clinical trial. J. Res. Med. Sci..

[43] Karimi-Nazari E., Nadjarzadeh A., Masoumi R., Marzban A., Mohajeri S.A., Ramezani-Jolfaie N., Salehi-Abargouei A. (2019). Effect of saffron (*Crocus sativus* L.) on lipid profile, glycemic indices and antioxidant status among overweight/obese prediabetic individuals: A double-blinded, randomized controlled trial. Clin. Nutr. ESPEN.

[44] Moravej Aleali A., Amani R., Shahbazian H., Namjooyan F., Latifi S.M., Cheraghian B. (2019). The effect of hydroalcoholic Saffron (*Crocus sativus* L.) extract on fasting plasma glucose, HbA1c, lipid profile, liver, and renal function tests in patients with type 2 diabetes mellitus: A randomized double-blind clinical trial. Phytother. Res..

[45] Rajaei Z., Hadjzadeh M.A., Nemati H., Hosseini M., Ahmadi M., Shafiee S. (2013). Antihyperglycemic and antioxidant activity of crocin in streptozotocin-induced diabetic rats. J. Med. Food.

[46] Yaribeygi H., Mohammadi M.T., Sahebkar A. (2018). Crocin potentiates antioxidant defense system and improves oxidative damage in liver tissue in diabetic rats. Biomed. Pharmacother..

[47] Shirali S., Zahra Bathaie S., Nakhjavani M. (2013). Effect of crocin on the insulin resistance and lipid profile of streptozotocin-induced diabetic rats. Phytother. Res..

[48] Razavi B.M., Hosseinzadeh H. (2017). Saffron: A promising natural medicine in the treatment of metabolic syndrome. J. Sci. Food Agric..

[49] Farkhondeh T., Samarghandian S. (2014). The effect of saffron (*Crocus sativus* L.) and its ingredients on the management of diabetes mellitus and dislipidemia. Afr. J. Pharm. Pharmacol..

[50] Nemati Z., Harpke D., Gemicioglu A., Kerndorff H., Blattner F.R. (2019). Saffron (*Crocus sativus*) is an autotriploid that evolved in Attica (Greece) from wild Crocus cartwrightianus. Mol. Phylogenet. Evol..

[51] Aleström P., D’Angelo L., Midtlyng P.J., Schorderet D.F., Schulte-Merker S., Sohm F., Warner S. (2020). Zebrafish: Housing and husbandry recommendations. Lab. Anim..

[52] Livak K.J., Schmittgen T.D. (2001). Analysis of relative gene expression data using real-time quantitative PCR and the 2(-Delta Delta C(T)) Method. Methods.

